# Structural and Molecular Kinetic Features of Activities of DNA Polymerases

**DOI:** 10.3390/ijms23126373

**Published:** 2022-06-07

**Authors:** Aleksandra A. Kuznetsova, Olga S. Fedorova, Nikita A. Kuznetsov

**Affiliations:** 1 Institute of Chemical Biology and Fundamental Medicine, Siberian Branch of Russian Academy of Sciences, 630090 Novosibirsk, Russia; fedorova@niboch.nsc.ru (O.S.F.); nikita.kuznetsov@niboch.nsc.ru (N.A.K.); 2 Department of Natural Sciences, Novosibirsk State University, 630090 Novosibirsk, Russia

**Keywords:** protein–DNA interaction, DNA polymerase, structural family, catalytic mechanism, kinetics

## Abstract

DNA polymerases catalyze DNA synthesis during the replication, repair, and recombination of DNA. Based on phylogenetic analysis and primary protein sequences, DNA polymerases have been categorized into seven families: A, B, C, D, X, Y, and RT. This review presents generalized data on the catalytic mechanism of action of DNA polymerases. The structural features of different DNA polymerase families are described in detail. The discussion highlights the kinetics and conformational dynamics of DNA polymerases from all known polymerase families during DNA synthesis.

## 1. Introduction

DNA polymerases play a key role in the maintenance of genome stability in living organisms. Enzymes of this class catalyze the polymerization of 2′-deoxyribonucleotides along a DNA strand, which the enzyme “reads” and uses as a template. The reaction proceeds with the participation of 2′-deoxynucleoside-5′-triphosphates (dNTPs), which are incorporated into the DNA strand as 2′-deoxynucleoside-5′-monophosphates (dNMPs), with a release of pyrophosphate (PPi). The type of a new nucleotide to be incorporated is determined by the principle of complementarity with the nucleotide located in the template that is being read. Thus, the newly synthesized molecule is fully complementary to the template strand and identical to one of the strands of the DNA double helix. This process is called *replication*. Its main principles are outlined in many reviews and textbooks on molecular biology and biochemistry. It should be noted that the structure of DNA polymerases is quite conserved; catalytic subunits of these enzymes differ little among living cells from different species. It is possible that such conservation of structure and low variation are due to the great importance, or even indispensability, of these enzymes for the functioning of cellular processes.

**Scheme 1:**
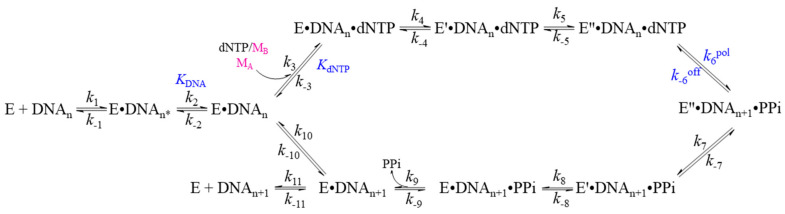
The kinetic mechanism of dNTP incorporation into a DNA primer by polymerase.

The kinetic scheme of the incorporation of nucleotides into the growing DNA primer chain under the action of DNA polymerases involves the participation of a divalent metal ion and the ordered addition of substrates (Scheme 1) [1,2,3,4]. In the first stage, the primer–template duplex binds to the polymerase, thereby forming the E•DNA_n*_ binary complex (where E = enzyme). In this process, the terminal base-pair can end up inside the active site of the polymerase, thus blocking dNTP binding. Translocation of the DNA within the complex gives rise to the E•DNA_n_ state with correct positioning of the 3′ terminus of the primer, and creates necessary space for incoming dNTP and its binding in the next step. This step also includes the association of a divalent metal ion. After the formation of “open” ternary complex E•DNA_n_•dNTP, many polymerases undergo conformational changes, which yield “closed” ternary complex E′•DNA_n_•dNTP [1,3]. The catalytically competent state E″•DNA_n_•dNTP arises after a second conformational change in the active site of the polymerase. Reactive groups, including divalent metal ions, catalytic carboxylate residues, 3′-OH in the primer strand, and the α-phosphate of the linked nucleotide are properly arranged for subsequent nucleotide transfer. As a result, the chemical stage proceeds, and the primer strand is extended by one nucleotide with the formation of the E″•DNA_n+1_•PP_i_ complex. After the chemical step, the enzyme returns to its original state, and pyrophosphate (PP_i_) is released from the active site of the polymerase. Next, the process can continue in two ways. Either the polymerase can be translocated by one base-pair (bp) along the DNA to carry out subsequent cycles of nucleotide incorporation (processive DNA synthesis), or the binary complex can dissociate (distributive DNA synthesis). Specific details of this model may depend on the DNA polymerase or system in question. The incorporation process and the order of elementary steps in Scheme 1 are supported by the kinetic, structural, and/or biophysical data reviewed in refs. [1,2,3,4].

**Figure 1:**
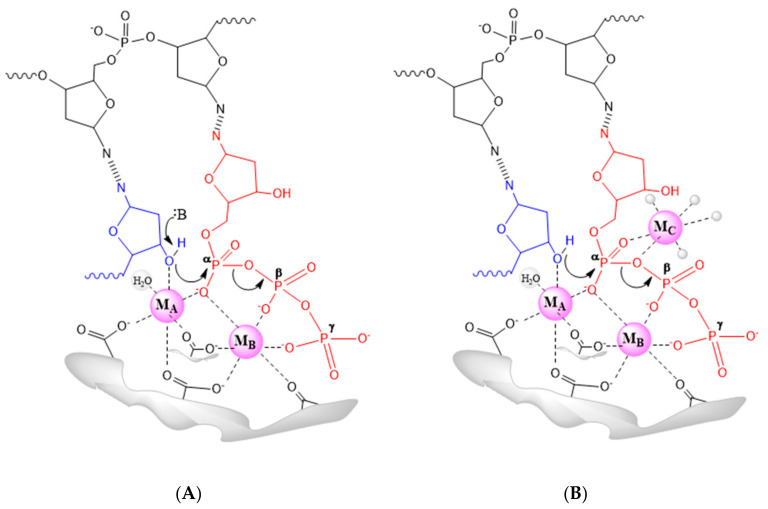
The proposed two-metal-ion mechanism (**A**) and three-metal-ion mechanism (**B**). The incoming nucleotide is colored red, template and primer DNA are colored black and blue, respectively, enzyme moieties are colored grey, metal ions are magenta.

A molecular mechanism underlying the addition of nucleotides by DNA polymerases has been proposed, on the basis of an almost-identical mechanism documented for the 3′ → 5′ exonuclease activity of DNA polymerase I [5,6]: the so-called two-metal-ion mechanism. After the binding of polymerase at the junction in the primer–template duplex, the incoming nucleotide is bound and positioned in the active site via Watson–Crick interactions with a base in the template, through intermolecular contacts with amino acid residues (aa) of the active site, and through coordination bonding with divalent metal ions. One metal ion binds between the terminal O3′ atom of the primer and the α-phosphate of the incoming dNTP, and is often referred to as metal A (M_A_) or the catalytic metal ion (Figure 1A). The second metal ion is coordination-bonded by the incoming dNTP through nonbridging oxygen atoms of α-, β-, and γ-phosphates and is often called metal B (M_B_) or the nucleotide-binding metal ion (Figure 1A). During catalysis, M_A_ serves as a Lewis acid to reduce the pK_a_ of the 3′-OH group in the primer, for cleavage and a subsequent direct nucleophilic attack on the α-phosphate of dNTP; this gives rise to a trigonal-bipyramidal pentacoordinate transition state that is stabilized by the coordination bonding of metal ions with oxygens of β- and γ-phosphate groups. This step causes the inversion of α-phosphate stereochemistry and a synchronized release of M_B_-coordinated leaving pyrophosphate. The M_B_ ion orients the triphosphate moiety of the bound nucleotide into a state suitable for catalysis and destabilizes the polymerase ternary complex. After the nucleophilic attack, M_B_ stabilizes the pentacoordinate transition state and neutralizes the negative charge on the leaving PP_i_ group (Figure 1A).

At present, along with the two-metal-ion mechanism, a “third-divalent-metal-ion mechanism” is actively discussed in the literature (Figure 1B) [2,7,8,9,10,11]. In this mechanism, the metal ion in site B is stably associated with the incoming dNTP and with the enzyme. The binding of Me^2+^ at site A aligns the reactive components—in particular, 3′-OH—and promotes its deprotonation. After reaction initiation but before the products are released, a third metal ion (M_C_) binds and stabilizes reaction intermediates. The function of the third divalent metal ion may be stabilization of the transition state, product release, or catalysis of the reverse reaction.

This review briefly summarizes the main structural and molecular-kinetic features of the functioning of DNA polymerases belonging to various structural families. The focus of our review is confined to structural and kinetic aspects of the reactions catalyzed by DNA polymerases. The review includes the differences and similarities of DNA polymerase families as well as brief insight into the techniques utilized for study DNA polymerases. For each polymerase family the typical well-studied members are described with emphasis on conformational dynamics.

## 2. Classification of DNA Polymerases

DNA polymerases have structure resembling a right hand. They contain three main subdomains: the “palm”, “fingers”, and “thumb”. DNA binding occurs in the cavity formed by these subdomains. The catalytic center is based on conserved amino acid residues of the palm subdomain. The “fingers” position a template in the active site and bind dNTPs, whereas the thumb binds DNA. Exonuclease activity is implemented by a domain located separately: at the N terminus of the enzyme. In all polymerase families, the palm is rather conserved, whereas the thumb and fingers can vary in structure [12]. Despite structural similarity, DNA polymerases have been categorized into seven families based on phylogenetic analysis and similarity of primary protein sequences: A, B, C, D, X, Y, and RT (Table 1) [13].

DNA polymerases of family A have replication and repair activities. A corrective function is performed by 3′ → 5′ exonuclease activity, i.e., removal—in case of an error—of an incorporated noncomplementary nucleotide, thereby increasing the fidelity of DNA synthesis. On the other hand, 5′ → 3′ exonuclease activity is physiologically responsible for the removal of ribonucleotide primers used for the replication of the lagging strand of DNA. Polymerases of this family have been found in eukaryotes (Pol γ, Pol θ, and Pol ν), bacteria (Pol I), and viruses (T7 DNA Pol) [4,14,15].

Family B polymerases, in contrast to the enzymes of family A, do not possess 5′ → 3′ exonuclease activity. Most of such polymerases found in eukaryotes are also devoid of the domain responsible for the 3′ → 5′ exonuclease activity. Nonetheless, in other taxa, 3′ → 5′ exonuclease activity is 1000 times higher than that of Pol I from *Escherichia coli*. Family B polymerases have been found in eukaryotes (Pol ζ, Pol α, Pol δ and Pol ε), bacteria (Pol II), archaea (DNA polymerase B), and viruses (DNA polymerase T4) [4,14,15,16,17,18].

The C family of polymerases consists of enzymes that are the main proteins responsible for chromosomal replication. They do not bear much resemblance to polymerases from families A and B. They are holoenzymes whose activity depends on interaction with at least 10 other proteins, forming a large multisubunit complex. These enzymes have been found in bacteria, and an example of such a polymerase is Pol III. Its α-subunit has DNA polymerase activity and is bound to the ε-subunit, which has 3′ → 5′ exonuclease activity [4,15,19].

Family D polymerases are present in Euryarchaeota, with Pol D being an example [4,20]. This enzyme is a heterodimer. The small subunit DP1 is responsible for 3′ → 5′ exonuclease activity, whereas the large subunit DP2 has polymerase activity. The DP1 subunit shares low but significant homology with eukaryotic DNA polymerase δ [21].

Polymerases of family X are small monomeric proteins. These enzymes are a part of the repair system in the cell. They are indispensable for such repair systems as base excision repair and nonhomologous end-joining (NHEJ). These enzymes have been found in eukaryotes (e.g., Pol β, Pol σ, Pol λ, and Pol µ), bacteria (Pol X), archaea (Pol X), and viruses (DNA polymerase of African swine fever virus) [4,14,22]. Members of this family have a structurally similar 8 kDa domain responsible for dRP lyase activity, but only Pol β and Pol λ possess an active 8 kDa domain.

Family Y polymerases have been found in bacteria, eukaryotes, and archaea. These enzymes can recognize and bypass various types of DNA damage, such as thymidine dimers. The fingers subdomain of family Y polymerases is smaller than that of replicative polymerases. In addition, all members of the Y family contain an additional subdomain (“little fingers”), which, by interacting with the DNA major groove, helps DNA synthesis to proceed through damage in DNA. The reduced size of the fingers subdomain is mostly responsible for the ability to go through thymidine dimers because the cavity formed by three catalytic domains can accommodate a thymidine dimer [23].

The family of reverse transcriptases (RTs) includes retroviral reverse transcriptases and eukaryotic telomerases. In the course of reverse transcription, retroviral reverse transcriptases interact with various nucleic acids (duplexes RNA/RNA, DNA/RNA, RNA/DNA, or DNA/DNA), thus converting a single-stranded viral RNA genome into double-stranded pro-viral DNA. Some retroviral reverse transcriptases function as dimers, such as those from human immunodeficiency viruses (HIV) 1 and 2, whereas others, such as the reverse transcriptase of Moloney murine leukemia virus (MoMLV), are monomeric. Nevertheless, both types contain polymerase and RNase domains for the cleavage of viral RNA during DNA synthesis [24]. Telomerases are also affiliated with the family of reverse transcriptases: they employ integral RNA as a template for telomere synthesis [25].

The differences and similarities between families are summarized in Table 2.

## 3. Methods for Studying the Mechanism of Action of DNA Polymerases

Structural methods make a great contribution to the understandding of enzymatic-catalysis mechanisms; however, they can only provide information about a certain fixed state of the enzyme and substrate at some point in the enzymatic process, for example, in a catalytic complex. For catalytically active enzymes, it is possible to obtain only a crystal of the free enzyme and a crystal of the complex of the enzyme with a reaction product. Targeted substitution of catalytically active amino acid residues in the enzyme helps to substantially deepen this kind of research and to crystallize a catalytically active complex, i.e., to capture the moment of the reaction when structures of the enzyme and substrate are fully ready for a catalytic event; however, the catalysis cannot take place because a functionally important amino acid residue is absent. There are several other approaches, which help to prepare the crystals of “intermediate” enzyme–substrate complexes, for example, by making a substrate modification that does not affect the binding to the enzyme but prevents the catalytic reaction. A considerable shortcoming of static structural data is the limited number of possible states of the enzyme–substrate complex that can be registered using such methods. Furthermore, X-ray diffraction data are not always confirmed under equilibrium conditions in solution, especially when covalent crosslinks between components of the complex are introduced to generate crystals of complexes. Moreover, even if the totality of processes that must occur for the assembly of a catalytic complex is known, the sequence of these events remains unclear: the nature of interactions in the initial stages of substrate recognition, and formation of which contact enables discrimination between a substrate and “nonsubstrate”, and which interactions ensure the specificity of the enzyme.

A “crystal-less” technique for determining structure—nuclear magnetic resonance (NMR) spectroscopy based on ^15^N, ^13^C, and ^2^H atoms—has undergone great development [26,27,28,29]. Cryo–electron microscopy (cryo-EM) has made a significant impact on the structural analysis of macromolecular structures at near-atomic resolution. This imaging technique is widely used for biological samples that are flash-frozen in the native environment at cryogenic temperatures [30,31,32].

Currently, there are many methods for determining the mechanism of action of DNA polymerases. Techniques involving radioactive or fluorescent labels, intercalating fluorescent dyes, Förster resonant energy transfer, nanopores, and surface plasmon resonance are among the most popular [33]. Photoaffinity labeling should be mentioned as a method of structural and functional analysis in solution. Photoaffinity labeling allows investigators to detect unstable complexes and auxiliary protein factors. This technique implies the use of DNA substrates, which bear photoreactive groups at certain positions. The photochemical properties of these groups are adjusted for the use of excitation near UV light, to avoid stimulation of the intrinsic photoreactivity of the nucleic acids and proteins. Upon photoirradiation, the generation of highly reactive species occurs, resulting in the direct covalent modification of adjacent molecules [34,35,36,37]. This approach has been successfully utilized to research the substrate-binding sites of RNA polymerase [38], of DNA polymerases [34,39,40,41,42], and of the complex of DNA polymerase α with primase [43].

In experiments based on radioactively labeled substrates, a minimal kinetic scheme has been determined for the incorporation of a single nucleotide into a growing DNA strand using the Klenow fragment of DNA polymerase I [Pol I(KF)] [44,45]. The combination of single-turnover and isotope-trapping experiments allowed quantitative evaluation of the kinetic scheme for specific DNA sequences. This technique makes it possible to investigate the covalent stages associated with the direct incorporation of nucleotides and does not provide information about conformational rearrangements of the interacting molecules.

The use of fluorescently labeled nucleotides allows researchers to study the kinetics of a polymerization reaction in real time. By means of fluorescently labeled DNA sequences, the dependence of DNA binding to Taq Pol and Pol I(KF) on temperature [46,47] and on ionic strength [48] has been characterized, and conformational transitions of Pol I(KF) upon inclusion of nucleotides have been studied [49]. With the help of fluorescently labeled nucleotides, the mechanism behind the choice of the correct nucleotide by Pol I(KF) has been researched [50], and an approach has been developed for quickly determining the constant of DNA binding to Pol I(KF) and the catalytic activity of Pol I(KF) [51]. It was shown that the active site of the Pol I(KF) is flexible, to tolerate the bulky nucleotide analogs; moreover, the fluorescence changes observed during misincorporation were substantially different from those observed during correct nucleotide incorporation, implying that the conformations adopted during correct and incorrect nucleotide incorporation are distinct.

Intercalating dyes are also used to study DNA polymerase activity [52,53] and for real-time PCR [53]. This method has a major limitation: many dyes inhibit the polymerization reaction by binding to DNA. This approach is generally used in a semi-quantitative way to determine relative rates of reaction.

Förster resonant energy transfer analysis is a powerful tool for studying various interactions between proteins and DNA. By means of Förster resonant energy transfer, the rate of nucleotide incorporation has been measured, the maximal rate of Pol I(KF) catalysis has been determined [54], conformational transitions in a polymerase molecule have been characterized [55], and the mechanism behind the selection of correct nucleotides has been established [56]. Single-molecule Förster resonance energy transfer (smFRET) microscopy is used for the investigation of functional coordination and large-scale conformational changes in enzyme–DNA complexes [57,58,59,60]. Optical trapping should be noted as well; it is a powerful single-molecule technique that is well suited to gain a greater degree of mechanistic insight into the underlying molecular process [61]. The coupling between chemical catalysis and translocation have been determined using optical tweezers [62].

The nanopore approach is employed to analyze individual DNA or RNA molecules and to study interactions of nucleic acids with enzymes and nucleic-acid-binding proteins [63]. Using the nanopore technique, the dependence of the lifetime of a DNA complex with Pol I(KF) on dNTP concentration has been studied [64].

Real-time surface plasmon resonance has been utilized to investigate interactions of eukaryotic DNA polymerase β with various DNA substrates, such as single-stranded DNA, double-stranded DNA with a blunt end, double-stranded DNA with a gap, and a DNA template–primer duplex containing various mismatches at different positions [65]. Quantitative characteristics of kinetics have been determined for the Pol I(KF) interaction with DNA duplexes containing one to three mismatches at the 3′ end of the primer [66].

It should be noted that an assay of the conformational mobility of an enzyme and substrate during their interaction is a difficult and nontrivial task that requires a wide range of methods, including mathematical processing of the data [67]. Table 3 lists key kinetic parameters, such as *K*_d_^DNA^, *K*_d_^dNTP^, *k*^pol^, and *k*^off^, that have been obtained for a number of polymerases from different families. As can be seen, the rate constants differ between families as well as inside a single family, suggesting the importance of the biological roles and functions of every unique DNA polymerase.

## 4. Family A DNA Polymerases

**Figure 2:**
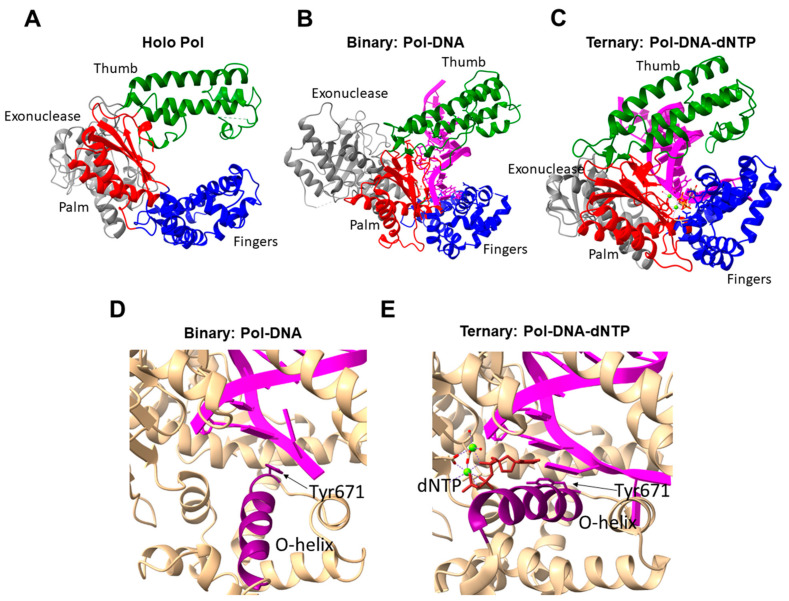
Three-dimensional structure of DNA polymerase I from *E. coli*: (**A**) holo enzyme (PDB ID 1ktq); (**B**) binary Pol-DNA (PDB ID 4ktq); (**C**) ternary Pol-DNA-dNTP (PDB ID 1qtm) complexes. The “thumb” and “fingers” subdomains are colored green and blue, respectively. The “palm” subdomain is red, “exonuclease” subdomain is grey, and DNA is magenta; (**D**,**E**) a detailed comparison of the active-site regions in the binary and ternary complexes. In each case, the protein is shown in beige, with the O-helix in purple. The incoming dNTP is colored in brown.

The first DNA polymerase to be characterized was Pol I DNA from *E. coli*. It is on the basis of kinetic and X-ray structural data on the Klenow fragment (and its homologs from bacteriophage T7 and thermophiles *Thermus aquaticus* and *Bacillus stearothermophilus*) that the mechanism of the DNA polymerase reaction (described above) was validated and the two-metal-ion mechanism was proposed [5,6,91]. When interacting with the enzyme, the DNA duplex “primer–template” binds in a shallow cleft between the thumb subdomain and 3′-exo domain. At the same time, a considerable part of the contact with the phosphate backbone is implemented by an α-helix of the thumb (Figure 2) [1,92]. In the polymerase–DNA binary complex, the terminal base-pair of the primer is adjacent to the region formed by a part of the “fingers” domain. This active-site boundary is formed predominantly by a long α-helix (O-helix) and extends throughout the fingers subdomain; it contains a set of important and highly conserved amino acid residues on the surface that are oriented inside the cleft. The side chain of a Tyr residue is at the C terminus of the O-helix. This Tyr residue is stacked with the terminal base-pair. When a correct dNTP is bound, a polymerase–DNA–dNTP ternary complex is assembled while the polymerase undergoes major conformational changes. During the transition from the open conformation (binary complex) to the closed conformation (ternary complex), the Tyr side chain of the O-helix C terminus moves further into the active site, away from its position, where it is stacked with the terminal base-pair. This arrangement enables the templating base to engage in a stacking interaction with its 3′ neighboring base at the end of the duplex and to form a base-pair with the incoming dNTP in a neatly fitting binding pocket, created by side chains of the O-helix and nearby residues on one side, and by the terminal base-pair of the primer on the opposite side.

**Figure 3:**
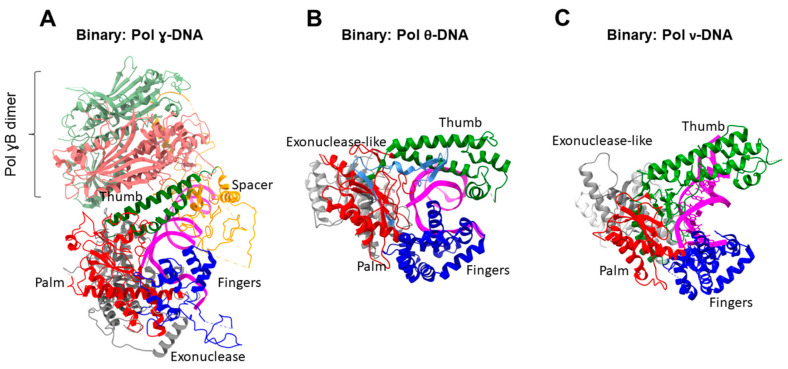
Three-dimensional structure of the binary complex of (**A**) Pol γ (PDB ID 5c52), (**B**) polymerase domain of Pol θ (PDB ID 4x0p), (**C**) Pol ν (PDB ID 4xvm) with DNA. The thumb (green), palm (red), fingers (blue), and exonuclease (gray) domains are presented. For Pol γ the spacer domain (orange) presents a unique structure. Structure of the heterotrimeric Pol γ holoenzyme contains one catalytic subunit and the Pol γB dimers (proximal (pink) and distal (light green) monomers).

In humans, the three members of the A family are known: Pol γ, Pol θ, and Pol ν (Figure 3). Pol γ is a high-fidelity mitochondrial polymerase that is responsible for replication and repair of the mitochondrial genome. In contrast, Pol θ and Pol ν are repair polymerases with low fidelity of the synthesis.

DNA polymerase θ consists of a C-terminal polymerase domain, a central domain, and an N-terminal domain. The enzyme lacks 3′ → 5′ exonuclease corrective activity and, therefore, has low fidelity of DNA synthesis. An additional loop in the catalytic center of the polymerase affords extra interactions with the primer, thus helping to stabilize a complex with a short primer and to extend DNA on a primer 2–3 nucleotides long. It is reported that Pol θ can act as a translesion polymerase, going past apurinic/apyrimidinic sites and adducts generated by reactive oxygen species. The functions of Pol θ in the cell have not been fully clarified. It was shown [93], that Pol θ can participate in base excision repair as a reserve polymerase in vivo. The involvement of Pol θ in the translesion repair of double-strand breaks during microhomologous end-joining has been demonstrated too [93].

The structure of the catalytic center of DNA polymerase ν differs from that of Pol θ in the unusual open state of the fingers domain; this conformation permits the binding of an incoming nucleotide, but not of its complementary base. How this specific feature affects the ability of Pol ν to go past lesions is still unclear [94].

## 5. Family B DNA Polymerases

In eukaryotic cells, three DNA polymerases of family B are responsible for nuclear-genome replication: Pol α, Pol δ, and Pol ε [95]. The DNA replication is initiated by a Pol α–primase complex on both the leading strand and lagging strand. The primase synthesizes short primers of 7–12 ribonucleotides (rNTPs), which are extended by Pol α by an additional 20–25 deoxyribonucleotides (dNTPs) [96]. After the primer synthesis, processive DNA synthesis is undertaken by Pol ε and Pol δ on the leading strand and lagging strand, respectively [97]. The contribution of Pol δ to genomic stability is not limited to high-fidelity DNA synthesis during replication. Pol δ probably takes part in most of the repair processes, although its exact function and extent of involvement still need further research [98,99].

**Figure 4:**
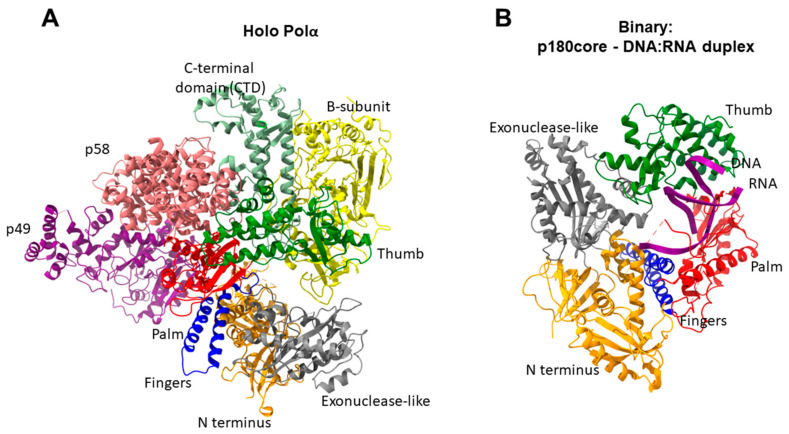
Three-dimensional structure of (**A**) the holo Pol α with primase (PDB ID 5exr), and (**B**) binary complex of catalytic subunit p180core with DNA–RNA duplex (PDB ID 4q5v). Pol α is composed of two polypeptides: the catalytic subunit (p180) and the accessory B-subunit (p70). p180 contains two domains, the catalytic (p180core) and the C-terminal (CTD, light green) domains. CTD connects the catalytic domain with B-subunit and primase, and contains two conserved zinc-binding modules. The p180core consists of five subdomains: N-terminal (orange), thumb (green), palm (red), fingers (blue) and exonuclease-like (gray) domains. DNA–RNA duplex is dark magenta. The N terminus of p180 is predicted to be poorly folded and does not participate in primer synthesis. Human primase consists of catalytic (p49, purple) and regulatory (p58, pink) subunits.

In vivo, eukaryotic replicative DNA polymerases are multi-subunit complexes (Figure 4) [95]. Replicative DNA polymerases have a similar core consisting of one large catalytic subunit and a smaller subunit, B. For example, in humans, it is p180–p70 for Pol α, p125–p50 for Pol δ, and p261–p59 for Pol ε. Each replicative DNA polymerase binds to a set of accessory subunits to form its heterotetrameric holoenzyme. For instance, the general human Pol δ (hPol δ) complex is associated with accessory subunits p66 (sometimes called p68) and p12 [100], whereas the general hPol ε complex interacts with accessory subunits p17 and p12 (in contrast to p12 alone for hPol δ) [101]. Nevertheless, in the case of hPol α, the p180–p70 nuclear complex enters into a heterotetrameric complex with a primase heterodimer that is composed of catalytic subunit p49 and regulatory subunit p58 [102]. Structures of *Saccharomyces cerevisiae* Pol α [103], Pol δ [104,105], and Pol ε [106] and of hPol δ [107] indicate that all three types of replicative DNA polymerase are characterized by a globular catalytic core and an extended structure—which is a part of the C-terminal domain of the catalytic subunit—as well as B- and auxiliary-subunits. These regions take part in the regulation of the coordination and dynamics of replicative DNA polymerases [107,108]. In the absence of the three small subunits, the catalytic subunit of each replicative DNA polymerase is capable of catalyzing template-dependent DNA synthesis in vitro. On the other hand, B- and accessory-subunits either regulate or enhance the DNA polymerization activity of the holoenzymes [106,109,110,111].

**Scheme 2:**
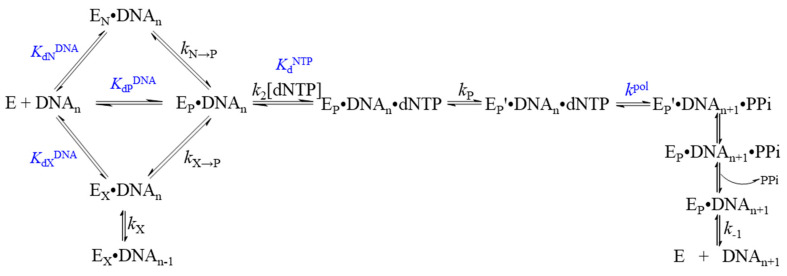
The kinetic mechanism of incorporation of the correct nucleotide as catalyzed by the holoenzyme of human DNA polymerase ε [71].

For human DNA polymerase ε, the influence of small subunits on DNA binding and on the kinetics of the polymerization reaction has been assayed [71]. It has been shown that the cooperation of p261C and small subunits leads to processive DNA synthesis [71,97,112]. After a pre-steady-state kinetic assay, the authors of ref. [71] concluded that hPol ε probably binds to DNA, with the formation of three different complexes after pre-incubation with a DNA substrate: a complex ready for a subsequent polymerization reaction (E_P_•DNA), an incompetent complex (E_N_•DNA), and a complex in which the DNA is bound in the 3′ → 5′ exonuclease center (E_X_•DNA; Scheme 2).

Although most researchers rely on the two-metal-ion mechanism in their data interpretation, there is evidence of possible participation of a third metal ion in the polymerization process. In ref. [99], an all-atom molecular dynamics simulation of several Pol δ DNA polymerases was conducted. It was demonstrated that during the emergence of a catalytically competent complex, multiple conformational changes and specific interactions occur that contribute to the function and fidelity of Pol δ. In particular, it was found that rotation in the fingers domain transforms Pol δ from an open to a closed conformation, thereby creating a well-organized active site only in the case of binding of the correct nucleotide and three magnesium ions. In the open state, tight bonds between the fingers domain and palm domain are reported that are designed to anchor the primer terminus in the active site. A loss of metal ion C disrupts active-site assembly and stimulates conformational changes in the thumb domain and in the conserved β-hairpin of the exonuclease domain, thereby inducing a transfer of the primer strand to the exonuclease domain for editing.

## 6. Family C DNA Polymerases

**Figure 5:**
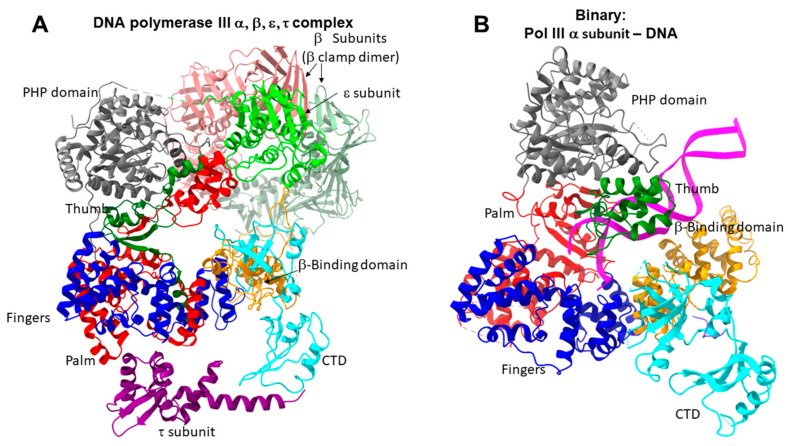
The cryo-EM structure of (**A**) the *E. coli* replicative DNA polymerase complex (DNA polymerase III alpha, beta, epsilon, tau) (PDB ID 5fku), and (**B**) the crystal structure of the alpha-subunit–DNA complex (PDB ID 3e0d). The holoenzyme is composed of 10 subunits (α, β, ε, θ, δ′, γ, τ, χ, and ψ), together with the helicase DnaB and the RNA primase DnaG form the replisome. Pol IIIα contains six domains with different functions: the N-terminal Zn^2+^-dependent 3′-5′ co-proofreading exonuclease polymerase and histidinol phosphatase (PHP) domain (gray); the catalytic palm domain (red); the incoming nucleotide-interacting fingers domain (blue); the nascent DNA gripping-thumb domain (green); the β-sliding clamp-binding domain (orange); and the CTD (cyan), which contains an oligonucleotide-binding OB fold and a possible external clamp-binding site at the extreme C terminus (cyan).

The DNA polymerases that replicate bacterial chromosomes belong to polymerase family C, which shares no sequence homology with any of the other DNA polymerase families. In Gram-negative bacteria, the replicative polymerase is the α-subunit of polymerase III (Pol III) (Figure 5), whereas in Gram-positive bacteria, the replicative polymerase is called Pol C. Although the first polymerase from family C was discovered as far back as 1971 [113], “C” is the last family of polymerases to be studied structurally [113,114,115,116,117]. Family C polymerases are rather large multidomain proteins, larger than polymerases of other families. A remarkable finding of crystallographic studies is that the bacterial replicative polymerase of this family is not related to either eukaryotic or archaeal replicative polymerases.

The structure of the active site of apo-enzyme Pol C from *Geobacillus kaustophilus* and apo-enzymes Pol III from *E. coli* and *Thermus aquaticus* [114,117] differs from that of canonical polymerases of families A and B, such as Pol I and Pol II, and from the active-site structure of eukaryotic replicative DNA polymerases Pol δ and Pol ε; the topology of apo-enzyme Pol C from *G. kaustophilus*, and of apo-enzymes Pol III from *E. coli* and *T. aquaticus*, is homologous to that of Pol β from family X. One of unique features of Pol III and Pol C is the extended fingers domain, which is much larger than that of any other known polymerase. The DNA duplex is held between the thumb domain and C-terminal domain of Pol C in the duplex-binding domain. A single-stranded DNA template enters the polymerase active site through the cleft formed between the “fingers” and duplex-binding domain. The oligonucleotide/oligosaccharide-binding domain (OB) (Pfam PF01336) and polymerase and histidinol phosphatase (PHP) domain (Pfam PF02811) are located at the N terminus of the polymerase domain [115]. In the ternary complex of Pol III [116], the oligonucleotide/oligosaccharide-binding domain seems to form a path directing the template strand to the active site. A short fragment of the downstream single-stranded DNA template occupies a cleft formed by the duplex-binding domain and fingers domain in Pol C, but does not reach the oligonucleotide/oligosaccharide-binding domain. The latter in Pol C is located where it can bind to the template strand 15–20 nucleotides ahead of the polymerase active site [115]. The PHP motif is widespread among family C polymerases but rare among other polymerases [118]. In Pol C, the PHP domain is a distorted (βα)7 barrel and engages in coordination bonds with up to three metal ions and phosphate. All metal-coordinating residues are highly conserved among Pol C proteins of different species, indicating functional importance for metal binding.

When Pol C binds to a DNA duplex, the latter has the standard B conformation, except for the end of the primer, where the ribose is in the C3-endo conformation required for an inline attack of 3′-OH on the α-phosphate of the incoming nucleotide [115]. The nascent base-pair is placed into a tight pocket consisting of a terminal base-pair and amino acid residues of the fingers and palm. The incoming nucleotide triphosphate is held in place by direct and water-mediated hydrogen bonds with Arg1238, Arg1218, and Tyr1269 in the fingers domain and with Lys970 and Ser895 in the palm, and via Metal B coordination. All residues within 4 Å from bound dGTP are strictly conserved among Pol C orthologs [115]. It is noteworthy that the interaction of Pol C with a DNA substrate extends far beyond the active site, with the thumb domain and duplex-binding domain making the greatest contribution to these interactions. Two highly conserved antiparallel β-sheets of the thumb (Tβ1–Tβ2) make hydrogen-bonding and van der Waals contacts with phosphodiester backbones of both strands in the minor groove at primer positions −3, −4, and −5, and at template positions −5 through −8. Such interactions with DNA are unique, and have not been documented for the structures of other DNA polymerases. Moreover, a part of the palm domain, together with an “index finger”, undergoes significant conformational alterations when binding to an incoming nucleotide. It should be pointed out that large (30°) rotation of the duplex-binding domain is observed during DNA binding [114,115,116]. This conformational flexibility of family C polymerases probably ensures spatial access for some repair polymerases to access the DNA and β-clamp.

**Scheme 3:**
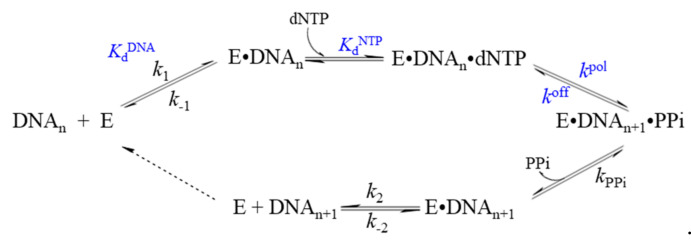
Minimal single-nucleotide incorporation reaction pathway for PolC (Sau-PolC-∆N∆Exo) from *Staphylococcus aureus* [79].

In ref. [79], steady-state and pre-steady-state kinetics of incorporation of a correct dNTP were characterized for a mutant Pol C polymerase (Sau-PolC-∆N∆Exo) from *Staphylococcus aureus*. The kinetic steps were found to follow the same pathway as that used by other polymerases, but Pol C has several distinctive characteristics (Scheme 3). The authors noticed an equilibrium process between the steps of nucleotide binding and the chemical step of the phosphoryl transfer reaction. They hypothesized that either the release of PP_i_ after the catalysis or a conformational change that precedes the release of PP_i_ can serve as a rate-limiting step in the catalytic cycle, thereby helping the polymerase maintain a conformation favorable for the reverse reaction. Perhaps this step enables a rudimentary corrective function of Pol C.

## 7. Family D DNA Polymerases

The polymerases of family D (Pol D) are considered the main replicative polymerases in archaea. Although Pol D behaves, in all respects, as a true polymerase participating in DNA replication, it differs structurally from all other known DNA polymerases. Pol D is a heterodimeric replicative DNA polymerase consisting of a large catalytic subunit (DP2) and a smaller subunit (DP1) that possesses corrective 3′ → 5′ exonuclease activity [119,120]. The activities of the two subunits are interdependent, and the presence of both subunits is required for the functioning of either of them; this arrangement is unique to the D family [121,122]. Based on crystal structures of catalytic nuclei of DP1 and DP2, it has been revealed that Pol D is an atypical DNA polymerase that has all the functional properties of a replicative DNA polymerase, but with an RNA polymerase catalytic core [123]. Pol D shares structural homology with the double-psi β-barrel (DPBB) family of RNA polymerases, where the catalytic center is formed between two DPBBs [124].

**Figure 6:**
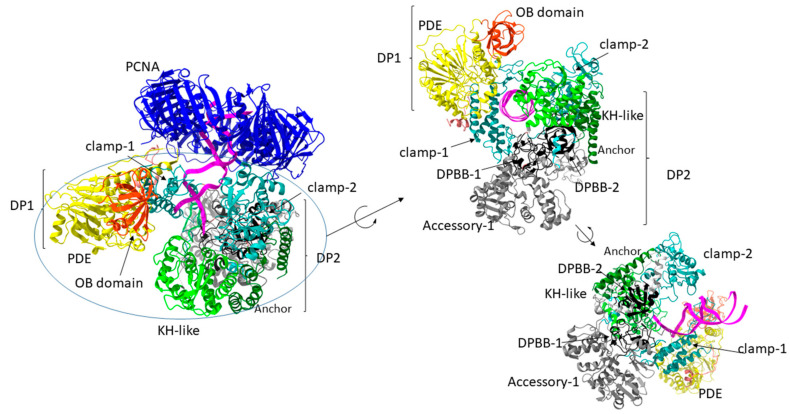
The cryo-EM structure of the DNA-bound Pol D-PCNA processive complex (PDB ID 6t8h). PCNA trimer is colored blue. Overview of Pol D domain organization: DP1 includes OB (orange) and PDE (yellow); DP2 includes DPBB-1 and DPBB-2 (black), clamp-1 and clamp-2 domains (cyan), KH-like (green)—which is connected to the Anchor domain (dark green)—and Accessory-1 and Accessory-2 domains (grey).

In ref. [125], the structure of the complex of *Pyrococcus abyssi* Pol D with DNA was determined using cryo–electron microscopy (Figure 6). The Pol D active site is claw-like and contains a catalytic core consisting of two DPBBs in the center and three zinc-binding modules (called Zn-I, Zn-II, and Zn-III domains) at the edges. The DNA substrate is nestled between two parts of the clamp domain, called 1 and 2, originating from DPBB-1 and DPBB-2, respectively. The clamp domain is bound on one side by a globular domain (aa 85–283) situated in the N-terminal region of DP2. The N-terminal domain of DP2 shares structural homology with the K-homologous (KH) domain of type II. Two accessory domains, named -1 and -2 (resulting from insertions within subdomains DPBB-1 and DPBB-2, respectively) play a structural role by combining the essential two-DPBB catalytic cores and the domains clamp-1 and clamp-2 into a scaffold. The KH-like domain is bound to the anchor domain, which strongly attaches it to clamp-2 and ensures its appropriate orientation within the active site.

The oligonucleotide-binding domain is located in an N-terminal region of the large Mre11-like nuclease-phosphodiesterase domain (PDE) of subunit DP1. The latter extensively interacts with DP2′s C-terminal region that is a part of clamp-1. This interaction is necessary for the rearrangement of the DP2 catalytic core into an active conformation. A comparison of the structure of the Pol D–DNA complex (as determined by cryo–electron microscopy) with the crystal structure of DP2 indicates that during the formation of the Pol D–DNA complex, there is an ordering of the DPBB-1 domain and its correct orientation in the final double catalytic center of DPBB; the clamp-1 domain and accessory-1 domain rotate by approximately 45° relative to the clamp-2 domain; and the KH-like domain moves ~10 Å from the catalytic site [125]. It must be mentioned that the Pol D catalytic core with two DPBBs differs from Klenow-like and Pol β-like structures. Additionally, Mre11-like nuclease DP1 differs from the DnaQ-like exonuclease domains found in most DNA polymerases that possess a corrective activity [126]. The KH- and clip-like DNA-binding domains of Pol D are structurally distinct from the palm, thumb, and fingers domains of other DNA polymerases.

In ref. [80], a kinetic analysis of Pol D from *Thermococcus* sp. 9° N was performed. It was shown that Pol D, indeed, follows the same general polymerization kinetic pathway as DNA polymerases from other families (see Scheme 1). The kinetics of pyrophosphorolysis indicated that the nucleotide incorporation is, indeed, reversible, with the maximal rates of nucleotide incorporation and *K*_d_(dNTP) being higher than the maximal rates of pyrophosphorolysis and *K*_d_(PP_i_); this state of affairs shifts the equilibrium toward nucleotide incorporation. Although Pol D is regarded as a replicative polymerase in Euryarchaea, the maximal polymerization rate (*k*_pol_) of Pol D is one of the slowest among kinetically characterized replicative polymerases (Table 3).

## 8. Family X DNA Polymerases

In eukaryotes, the X family of polymerases is composed of DNA polymerases β, λ, and μ, and terminal nucleotidyl transferase TdT, which are components of the cell’s repair system. DNA polymerases β, λ, and μ can also participate in translesion DNA synthesis. They are essential for such repair systems as base excision repair (BER) and non-homologous end-joining (NHEJ) [22,127]. Base excision repair is the main process that removes specific damage to nitrogenous bases that is caused by reactive oxygen species, alkylating agents, and some other factors. It removes at least 20,000 lesions per cell per day. Pol β is a key protein in base excision repair. NHEJ serves to repair DNA double-strand breaks. At the first step of the process, the break is recognized by proteins Ku70/80, which binds to the DNA at the break, thereby preventing the ends from separating. After that, the protein is translocated so that the ends of the double-strand break become physically free for other enzymes. Then, Ku70/80 involves the DNA-dependent protein kinase (DNA-PKcs) and the Artemis protein in the process; they prepare the ends of the break for subsequent ligation (via endonucleolytic activity of the Artemis protein) and bring the ends together. The resultant single-strand breaks are filled by family X polymerases (Pol λ, Pol μ, or TdT). In the last stage, the sugar–phosphate backbone is stitched up by a ligase.

The best-studied member of the X family is DNA polymerase β (Pol β). This is the smallest DNA polymerase in the cells of living creatures. It consists of 335 aa, amounting to 39 kDa. In Pol β, as in other polymerases of the X family, there is no 3′ → 5′ exonuclease corrective activity; therefore, the accuracy of synthesis low. Pol β includes a lyase domain, catalytic (palm) domain, nucleotide-binding (fingers) domain, and DNA-binding (thumb) domain.

**Figure 7:**
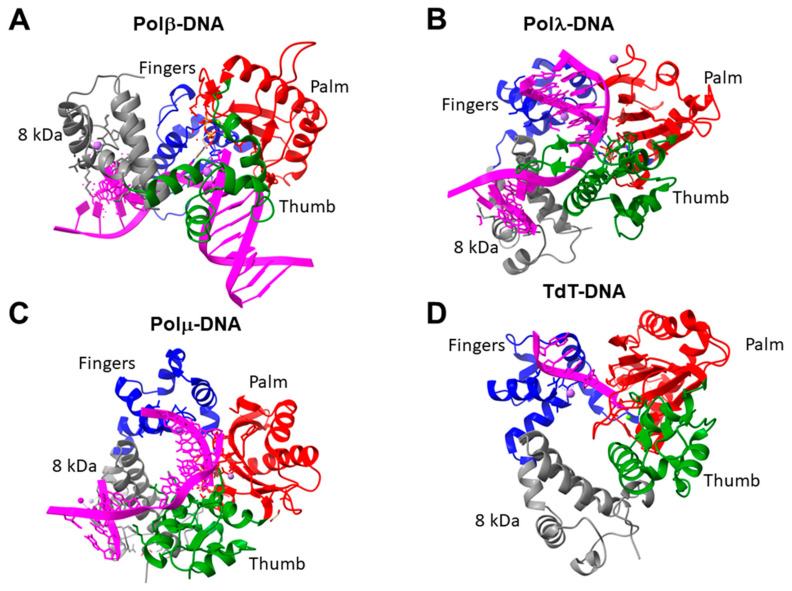
Three-dimensional structure of the binary complex of (**A**) Pol β (PDB ID 1bpy), (**B**) Pol λ (PDB ID 1xsn), (**C**) Pol µ(PDB ID 5tyw), and (**D**) TdT (PDB ID 4i2a) with DNA. The thumb (green), palm (red), fingers (blue) and exonuclease (gray) domains are presented.

X-ray diffraction data on Pol β in complex with a DNA substrate containing a gap or break show that the DNA is bent by 90° (Figure 7) [128]. In the enzyme–DNA binary complex, Pol β is in an open conformation. Upon binding of a correct dNTP, the fingers subdomain rotates by approximately 30° around α-helix M, thus assuming a closed conformation, affording a catalytically competent state. In the enzyme–DNA–ddCTP ternary complex, the 8 kDa domain ends up near the fingers domain, thereby causing the enzyme to take the shape of a doughnut [128]. dNTP binding involves the fingers subdomain, which shifts by ~12 Å during the transition between the open conformation (binary complex) and closed conformation (ternary complex) [128]. On the basis of crystal structures of Polβ with various substrates, amino acid residues that are key to various polymerase functions have been identified [128,129]. Residues Arg283 and Tyr271 are important for the protein–template interaction: in the closed conformation, a hydrogen bond emerges between Arg283 and the nucleotide located in the template opposite the terminus of the primer in the DNA minor groove; in the open conformation, a hydrogen bond forms between Tyr271 and a base in the template. Tyr271 also engages in hydrogen bonds with the primer terminus in the minor groove, while the helix–hairpin–helix (HhH) motif in a thumb subdomain (aa 92–118) binds the primer backbone. Another HhH motif in the 8 kDa domain (aa 55–79) interacts with the DNA substrate strand downstream of the gap or nick. The dNTP-binding pocket, which contains residues Tyr271, Phe272, Asp276, and Asn279, drives the interaction of the enzyme with dNTP by creating hydrogen bonds between Asn279 and the incoming nucleotide in the minor groove of DNA.

**Scheme 4:**
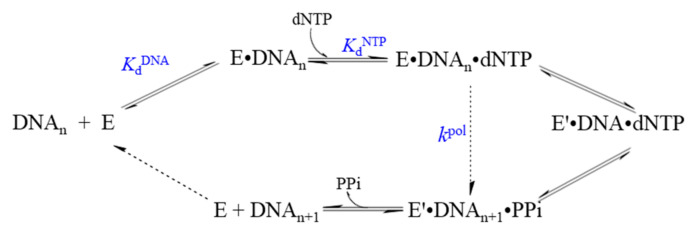
Minimal single-nucleotide incorporation reaction pathway for Pol β and Pol λ.

The catalytic mechanism of action of Pol β involves the ordered binding of substrates (Scheme 4). First, Pol β associates with DNA, with the preferred substrate being DNA containing a short gap with 3′-OH and 5′-phosphate. This event is followed by the binding of dNTP, which enters into hydrogen bonds with a base in the template. When dNTP binds, the enzyme undergoes conformational changes that include global subdomain movements and minor rearrangements of side chains. In the enzyme, such conformational alterations position substrates for a direct nucleophilic attack of O3′ on the α-phosphate of the incoming nucleotide, according to the two-metal-ion mechanism. Residues Asp190, Asp192, and Asp256 bind two magnesium cations (Metals A and B). Metal A interacts with the incoming nucleotide, giving rise to a tridentate structure. Metal B engages in a coordination bond with oxygen at the 3′ end of the primer and oxygen in the α-phosphate group of the incoming nucleotide. After the nucleotidyl transfer, a second conformational change possibly happens, including the opening of a subdomain, allowing for the release of the pyrophosphate and of the reaction product [22,129].

DNA polymerase λ (Pol λ) was discovered in the year 2000; it stands out among other polymerases of the X family, because Pol λ has all the enzymatic functions that are related to the X family: DNA polymerase, transferase, and dRP-lyase activities. The presence of several enzymatic activities enables this enzyme to participate in various processes: base excision repair, double-strand break repair, V(D)J recombination, and translesion synthesis. Pol λ contains an N-terminal BRCT domain (which plays an important part in protein–protein interactions of the enzyme), a serine-and-proline-rich region (where post-translational modifications occur), and a C-terminal domain (catalytic region). The catalytic domain consists of an N-terminal 8 kDa lyase subdomain and a polymerase subdomain. It is worth noting that Pol λ prefers Mn^2+^ to Mg^2+^ as a cofactor. Furthermore, Pol λ synthesizes DNA more efficiently from an RNA primer, and can add either dNTP or rNTP [130]. In contrast to Pol β, large-scale domain movements are not observed after Pol λ binding to a correct dNTP [131]. For Pol λ, correct positioning of the DNA template strand is important. In the Pol λ–DNA binary complex, the template strand is displaced by approximately 5 Å outward from the DNA binding cleft. In this context, the overall conformation of the protein is more reminiscent of the Pol β ternary complex, where an α-helix of the N domain is close to the active center. When a nucleotide binds to form a ternary complex, the template strand moves into the correct position. A comparison of the binary and ternary complexes of Pol λ indicates that the positions of catalytic residues Asp427, Asp429, and Asp490 do not depend on the presence or absence of the incoming nucleotide. The main structural alterations are related to residues Tyr505 and Phe506 (YF motif), which are shifted, making contacts in the minor groove with the correctly positioned DNA duplex [132]. This closed conformational state maintained by the 8 kDa lyase domain has been reported to play an important role in NHEJ [131]. The overall kinetic scheme for Pol λ is similar to that for Pol β [83,133].

Pol μ is a small protein with a molecular weight of 55 kDa and contains three domains: an N-terminal BRCA domain, an 8 kDa domain, and a C-terminal polymerase domain. The 8 kDa domain does not possess lyase activity, but is important for the ligation of 3′ protruding noncomplementary ends during double-strand break repair. The polymerase domain, aside from three catalytic subunits, contains an additional loop (loop I), which is present in TdT too. This loop allows Pol μ to act as a terminal transferase that attaches nucleotides to a single-stranded primer in the presence of Mn^2+^. Pol μ is also capable of attaching either dNTP or rNTP and is key to the repair of double-strand breaks by NHEJ. The ability of Polμ to perform DNA synthesis on a partially noncomplementary primer helps to ligate the termini of two DNA strands in the course of NHEJ.

X-ray diffraction data on the ternary complex of Pol μ with DNA and dNTP suggest that the enzyme binds tightly to the DNA template while DNA is bent by 90° in the active site of Pol μ [134]. This structure has several differences from the structures of Pol β and Pol λ. For instance, Polµ contains several flexible loops. One of them (aa 465–471) is located in the C-terminal domain and is much shorter than the homologous loop in Pol λ, which is responsible for stabilizing an extrahelical nucleotide present in frameshift intermediates of this fragment. Additionally, this loop in Pol μ is located much further from the DNA-binding site and is more similar in its conformation to the loop present in TdT. Loop 1 (situated between β-strands 3 and 4) in Pol μ is longer than similar loops in polymerases β and λ. Furthermore, it has ordered structure [134]. In terminal transferase, this loop is located within the substrate-binding cleft and is believed to facilitate the template-independent synthesis that is catalyzed by this enzyme. In Pol μ, deletion of this loop reduces the template-independent activity [135]. The His329 residue likely facilitates the template-independent synthesis by stabilizing the primer strand. Pol μ has fewer interactions with incoming dNTP than do polymerases β and λ, and this property may partly explain the ability of Polμ to incorporate ribonucleotides into the substrate. Just like Pol λ [131], Pol μ remains in the closed conformation throughout the catalytic cycle [136].

The mechanism of the polymerase reaction of Pol μ is similar to that of Pol β and Pol λ, but Pol μ is a distributive polymerase [137,138], with the DNA dissociation rate constant much higher than the polymerization reaction rate constant [83]. Under single-turnover conditions, the rate of polymerization of a correct dNTP substrate is significantly lower than that of Pol β and Pol λ. It must be mentioned that time-resolved crystallographic analysis of polymerases β [139,140,141,142,143] and μ [144] points to the possible participation of a third metal ion in their catalytic reaction.

Terminal deoxynucleotidyl transferase TdT conducts efficient template-free DNA synthesis. TdT takes part in V(D)J recombination by adding 1–10 nucleotides to the free 3′-OH terminus of the V segment, thus increasing immunological heterogeneity [145]. TdT, just like Pol λ [131] and Pol μ [136], stays in a closed conformation throughout the catalytic cycle [146]. TdT and Pol μ are more similar to each other than to Pol β or Pol λ. A unique feature of TdT is a specific loop (Loop1, consisting of 20 aa (positions 382–401) and located between the β3- and β4-chains) that prevents the binding of the 5′-overhanging end of the DNA template strand. Currently, information about more than 30 TdT structures (a wild-type or mutants in various complexes) is available in the Protein Data Bank, and in all of them, Loop1 adopts the same lariat-like conformation that prevents the template strand binding to TdT. In [147] a series of high-resolution X-ray structures simulating pre-catalytic, post-catalytic, and catalytically competent states of TdT are described. The effect of metal ions Mg^2+^, Mn^2+^, Co^2+^, and Zn^2+^ on the structure of the complexes was investigated in that study, and an active role of metal ions in the catalytic cycle was demonstrated. It was revealed that during the catalytic cycle of TdT, the conformation of the ribose residue switches from C2′-endo to C3′-endo, which is necessary for the direct attack of a deprotonated 3′-OH group on a phosphodiester bond (in the presence of Mn^2+^ or Mg^2+^ ions). In addition, the binding of the metal ion in the A site proved to be transient; metal ion A dissociates immediately after the chemical step and binds back after DNA translocation and the binding of a new dNTP. A similar mechanism has been described for single-subunit RNA polymerase [148]. Those authors propose the presence of a third metal-binding site (site C), into which the metal ion is “transferred” from binding site A immediately after the catalytic reaction; then, the ion probably transfers back after DNA translocation and dNTP binding. Nonetheless, this metal–C binding site turned out to be located at a distance of 18 Å from the metal A site between Loop1 and the SDR2 region [147]; this arrangement is very different from X-ray diffraction data about Pol β and Pol μ. Details of the kinetic mechanism for TdT, including the rate constants of the elementary steps of the process, remain unclear. The absence of a template strand should certainly affect the kinetic parameters for the steps of DNA and nucleotide binding, for the catalysis of the polymerization reaction, and for the dissociation of the product.

## 9. Family Y DNA Polymerases

**Figure 8:**
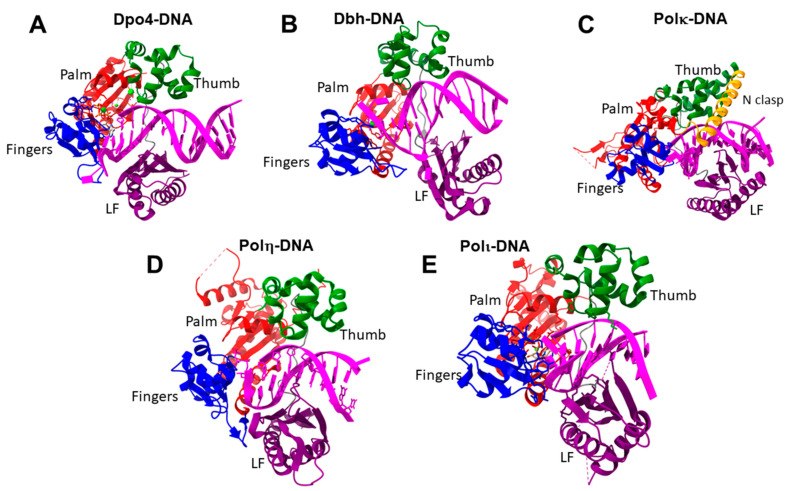
Three-dimensional structure of the complex of (**A**) Dpo4 (PDB ID 2agp), (**B**) Dbh (PDB ID 3bq1), (**C**) Pol κ (PDB ID 3pzp), (**D**) Pol η (PDB ID 4ed8), and (**E**) Pol ι (PDB ID 3gv8) with DNA. The thumb (green), palm (red), and fingers (blue) domains are presented; for Pol κ, the unique N-clasp domain at the N-terminus is presented (yellow).

The polymerases of family Y are classified into six major subfamilies based on amino acid sequences (Figure 8). This family is represented by the polymerases pol IV (known as DinB) and pol V (catalytic subunit is called UmuC) from *E. coli*, and the four human enzymes Pol η, Pol ι, Pol κ, and Rev1 [149]. All polymerases of the Y family are composed of two functional regions: the catalytic polymerase domain, which consists of 350–500 aa, and the regulatory region, which is 10 aa (for DinB, Dbh, and Dpo4) to 600 aa (for Rev1). Among these six subfamilies, bacterial DinB and its archaeal homologs Dpo4 and Dbh are related to eukaryotic enzyme Pol κ [149]. Pol η and Pol ι were originally thought to be close homologs, but it turns out that the two enzymes have only low functional similarity [150]. The catalytic domain of Y-family polymerases contains a conserved catalytic core that includes the fingers subdomain, palm subdomain, and thumb subdomain, as well as an additional “little finger” (LF) subdomain [149]. The latter, also known as the polymerase-associated domain (PAD), is unique to the Y family and shows greater sequence variation than the catalytic core does [149,151]. DNA binds between the thumb and LF subdomains. In contrast to the polymerases of other families, the catalytic domain of Y-family polymerases is a pre-formed active site with a closed conformation of the finger subdomain, even in the absence of DNA, dNTP substrate, or both [151]. Some members of the Y family can bind incoming dNTP, even in the absence of a template and primer [152]. It should be noted that the active site of these polymerases is rather large and solvent-accessible [149]. As a consequence, no big movement in the catalytic domain [153] is either required or observed during the complete cycle of the polymerization reaction. Such a “loose” fit of substrates in polymerases of the Y family leads to low accuracy, low catalytic efficiency, and low processivity of DNA synthesis. On the other hand, these properties help these polymerases to carry out DNA synthesis on a template containing bulky lesions.

Some members of the Y family are reported to undergo large conformational changes after binding to a substrate. The catalytic core and LF subdomain are connected via a flexible linker in all Y-family polymerases. In the DinB subfamily, e.g., in Dpo4, Dbh, and Pol κ, these domains move freely, relative to each other, in the absence of DNA [151,154,155]. In this case, DNA binding induces considerable conformational changes in the enzyme molecule [151,154]. Because interactions between the catalytic core and the LF subdomain are rather limited in Dpo4, Dbh, and Pol κ, the spatial separation of these domains is observed even in ternary complexes with DNA and dNTP [149]. In Pol κ, the catalytic core and LF domain interact through an N-terminal extension (N-clasp) [155]. The likely purpose of such spatial separation of domains in Dpo4, Dbh, and Pol κ is to accommodate a bulky lesion [156,157], or damage that generates a loop in a template DNA strand and results in a deletion frameshift [158]. Interactions between the catalytic core and LF domain in Pol η give rise to a “molecular splint” that retains the template strand in the B form even in the presence of a UV-crosslinked cis–syn pyrimidine dimer [158].

When researching the mechanism of the polymerase reaction in the Y family, investigators rely on the standard scheme of the mechanism widely accepted for polymerases [86,87,88,89]. The analysis of time-resolved X-ray diffraction data for hPol η suggests that a third magnesium ion participates in the product formation stage; moreover, it is probable that it not only stabilizes the leaving pyrophosphate group, but also that it is necessary for product formation [8,23,159].

## 10. DNA Polymerases of RT Family

Retroviral replication requires the conversion of single-stranded genomic RNA to double-stranded proviral DNA, which is later integrated into a host chromosome. Reverse transcription is performed by an enzyme called reverse transcriptase (RT). This multifunctional enzyme possesses activities of both RNA-dependent and DNA-dependent DNA polymerases and the activity of ribonuclease H (RNase H), which specifically cleaves the RNA strand of RNA/DNA hybrids. In mature virus particles of HIV-1, reverse transcriptase is present as a heterodimer consisting of subunits p66 and p51. Subunit p51 is derived from protease-mediated cleavage of subunit p66 between residues Phe440 and Tyr441, and lacks the RNase H domain [24].

**Figure 9:**
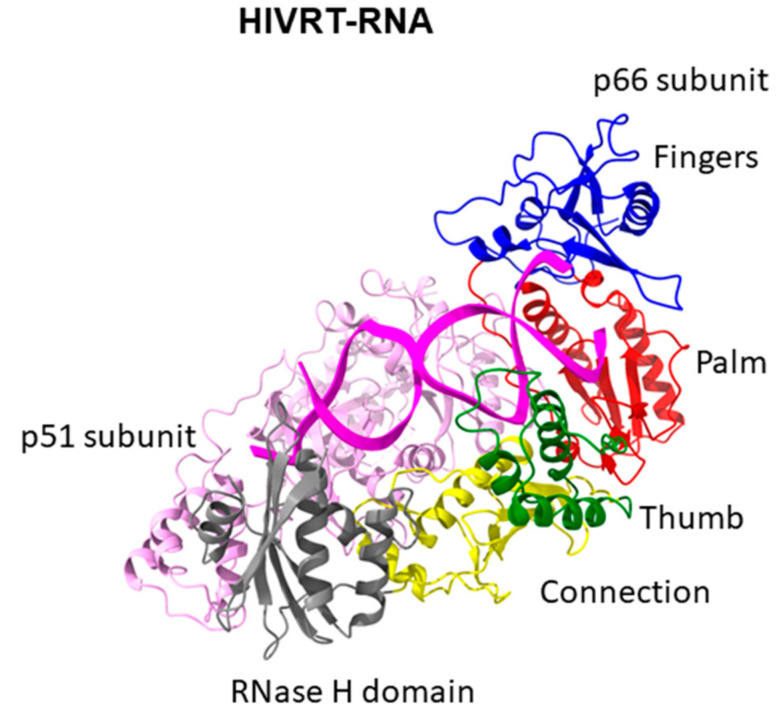
Three-dimensional structure of HIV-1 reverse transcriptase (RT) heterodimer in complex with a double stranded RNA (PDB ID 6hak). The thumb (green), palm (red), fingers (blue), RNase H (grey), and connection (yellow) domains are presented.

Just like other DNA polymerases, reverse transcriptase contains the fingers, thumb, palm, and junction subdomains (which are present in both p66 and p51) as well as an RNase H domain (identified at the C terminus of subunit p66; Figure 9) [160]. Although the tertiary structures of the four subdomains are similar between p66 and p51, their relative orientation toward each other differs between these subunits. As a consequence, unlike p66, p51 does not have the cleft necessary for the binding of the primer–template duplex. The crystal structure of a complex of HIVRT with a DNA–DNA duplex (primer–template) shows that the fingers, palm, and thumb of p66 form a nucleic-acid-binding cleft [161]. This binding groove—into which the duplex is folded by the fingers and thumb—can accommodate approximately 17–18 bp. The 3′ terminus of the primer is located in close proximity to the active site of the polymerase, and the last base-pair is situated near the active site of the RNase H domain. The crystal structure of a ternary complex of HIVRT with a DNA–DNA duplex and incoming dNTP indicates that conformational alterations in p66 proceed during the formation of a catalytic complex, and that the fingers subdomain bends to trap the incoming dNTP. As a result, a catalytically competent closed complex comes into being, in which the 3′-OH group of the primer’s end can attack the α-phosphate of dNTP. The fingertips can fold back somewhat before the next chemical step to release the pyrophosphate and facilitate the binding of the next dNTP [162]. The DNA duplex has an A-like conformation near the active site of the polymerase, but with a distinctly wider major groove and smaller base-pair tilt than the standard A conformation. It can increasingly become B-like in the upstream region, the major groove remains unusually deep for B DNA, and the base-pairs retain some tilt with respect to the helix axis. The two structurally distinct segments are separated by a kink (about 40°) around the seventh base-pair. Most of the interactions between the reverse transcriptase and the duplex take place near the active site of the polymerase and, albeit to a lesser extent, near the active site of the RNase H domain. The polymerase domain is in contact with the first 9 bp. Further upstream, a sequence of ~5 bases in the template strand stays mostly solvent-accessible until the template comes into contact with the RNase H domain. The latter domain mediates an interaction with ~4 bases. The reverse transcriptase binds to different primer–template combinations in the same way.

In [163], the experimental and theoretical analyses of the role of structural changes of HIVRT in the selection of the correct dNTP are presented. It is demonstrated that it is the rate of conformational changes—along with the affinity of the nucleotide for the open state—that determines the specificity constant for the selection of the correct dNTP. According to the predicted structure of the transition state, the authors hypothesize that long-range electrostatic interactions cause rapid collapse of the transition state into the final closed state, where other interactions (such as hydrogen bonds) contribute to overall stability and specificity.

**Scheme 5:**
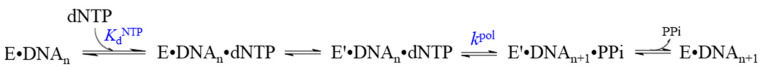
Minimal single-nucleotide incorporation reaction pathway for HIVRT.

Kinetic research on HIVRT has shown that this polymerase follows the same general polymerization kinetic pathway as do DNA polymerases from other families [90]. It is reported that the specificity constant (*k*_cat_/*K*_M_) is determined by the rate of substrate-induced conformational change [164,165] (Scheme 5). The binding of a nucleotide to an enzyme–DNA complex (E•DNA_n_, where DNA is *n* nucleotides long) proceeds in two steps. Initial weak rapid equilibrial binding is followed by rapid isomerization from the open state (E•DNA_n_•dNTP) to the closed state (E′•DNA_n_•dNTP), with a subsequent slower chemical reaction (characterized by *k*^pol^).

## 11. Conclusions

DNA polymerases perform crucial functions in the maintenance of genome stability by participating in such processes as repair, replication, and recombination. The molecular events underlying these processes are elucidated through the determination of rate and equilibrium constants of all individual reactions taking part in the polymerization cycle; these include DNA and dNTP binding, conformational changes, phosphoryl transfer, and kinetic steps related to the product release. Conformational dynamics make an important contribution to the high fidelity of DNA polymerases by ensuring selectivity based on the initial recognition of the canonical structure of the incoming base, and then by means of a transition state involving more substantial conformational changes in the enzyme. In this context, the incoming nucleotide enters into electrostatic interactions with charged amino acid residues, thus facilitating the transition of the complex to a closed state. These interactions depend on the correct geometry of base-pairs; this, in turn, is predicated on the correct hydrogen-bonding interactions and the correct positioning of base-pairs between the incoming nucleotide and a relevant base in the template. The network of long-range electrostatic contacts observed in the transition state rapidly becomes more ordered, only when the correct base-pair is present, thereby resulting in a quick transition to the closed state.

## Figures and Tables

**Table 1:** Families of DNA polymerases [13].

Family	Taxon	DNA Polymerases	Functions
A	Eukaryota Bacteria Viruses	Pol γ, Pol θ, and Pol ν Pol I T7 DNA Pol	Replication, repair
B	Eukaryota Bacteria Archea Viruses	Pol ζ, Pol α, Pol δ, Pol ε Pol II DNA pol B T4 DNA Pol	Replication, repair
C	Bacteria	Pol III	Replication
D	Archea	Pol D	Replication
X	Eukaryota Bacteria Archea Viruses	Pol β, Pol σ, Pol λ, Pol µ, TdT Pol X Pol X ASFV DNA Pol	Repair
Y	Eukaryota Bacteria Archea	Rev1, Pol ι, Pol κ, and Pol η Dbh, Pol IV and Pol V Dpo4 DNA Pol	Translesion synthesis
RT	Eukaryota Viruses	Telomerase Reverse transcriptase	RNA-dependent DNA synthesis

**Table 2:** The differences and similarities between DNA polymerase families.

Family	Polymerase Domain	Special Domains	Other Activities Present in DNA Polymerase
A	The catalytic domain includes palm, fingers, thumb subdomains	Exonuclease domain	3′ → 5′ exonuclease corrective activity (for most members); 5′ → 3′ exonuclease activity
B	Multi-subunit complex, catalytic core includes palm, fingers, thumb subdomains	CTD (responsible for connection of the catalytic domain with B-subunit and primase)	3′ → 5′ exonuclease corrective activity (devoid for most members from eukaryotes); primase activity (DNA synthesis de novo)
C	Large multidomain proteins, catalytic core includes palm, fingers, thumb subdomains	PHP domain; β-sliding clamp-binding domain; CTD containing an oligonucleotide-binding fold	3′ → 5′ exonuclease corrective activity
D	The heterodimeric polymerase consisting of DP1 and DP2 subunits	PDE domain; clamp-1 and clamp-2 domains; DPBB-1 and DPBB-2 domains; KH-like domain	3′ → 5′ exonuclease corrective activity
X	Small proteins, catalytic core includes palm, fingers, thumb subdomains	8 kDa domain; BRCT domain (important for protein–protein interactions)	dRP-lyase activity; single-strand DNA extension (for Pol µ, TdT)
Y	The catalytic core includes palm, fingers, thumb subdomains	regulatory region; little fingers domain	translesion DNA synthesis
RT	Heterodimeric polymerase consisting of two subunits, catalytic core includes palm, fingers, thumb subdomains	RNase H domain; connection domain	RNA template-dependent DNA polymerase activity; ribonuclease H activity

**Table 3:** A comparison of kinetic parameters of various polymerases.

Polymerase	Family	*K*_d_^DNA^, (nM)	*K*_d_^dNTP^, (µM) Correct N	*k*^pol^, (s^−1^)	*k*^off^, (s^−1^)	Ref.
Pol I (Klenow)	A	5	5.5	50	0.2	[44]
DNA polymerase T7	A	23	18	287	0.2	[68]
Pol T4	A	70	20	400	6	[69]
Human Pol α	B	58	9.2	26.8	7.0	[70]
Human Pol ε	B	22	11	411	ND	[71]
*E. coli* Pol II	B	21	4.4	13.1	0.05	[72]
Mammalian Pol δ	B	300	0.93	13	ND	[73]
Yeast Pol δ	B	30	24	0.93	0.03	[74]
Human mitochondrial large subunit Pol γ Holo Pol γ	B	39 9.9	14 0.78	3.5 45	0.03 0.02	[75] [76]
Vent Pol B	B		70	66	1.1	[77]
RB69	B		69	200	0.35	[78]
Sau-PolC-∆N∆Exo	C	390	4	180	150	[79]
Pol D	D		0.9–2.5	1.8–3.1	0.4	[80]
Pol β	X	49	110	10	0.3	[81]
Pol λ	X	0.15	1.1–2.4	3.0–6.0	ND	[82,83]
Pol µ	X		0.35–1.8	0.006–0.076	ND	[84]
*E. coli* Pol IV	Y	50	441	12	0.18	[85]
*Sulfolobus solfataricus* Dbh	Y	60	600	0.64–5.6	ND	[86]
*S. solfataricus* Dpo4	Y	10–40	70–230	7.6–16.1	ND	[87,88]
Yeast Pol η	Y	16	6.8–15	3.9–15.6	ND	[89]
RT	RT	4.7	4–14	33–74	0.16	[90]

ND: not determined.

## Data Availability

No applicable.
